# Transcriptome-Wide Analysis Revealed the Potential of the High-Affinity Potassium Transporter (*HKT)* Gene Family in Rice Salinity Tolerance via Ion Homeostasis

**DOI:** 10.3390/bioengineering9090410

**Published:** 2022-08-23

**Authors:** Shahid Hussain, Rui Zhang, Shuli Liu, Rongkai Li, Yicheng Zhou, Yinglong Chen, Hongyan Hou, Qigen Dai

**Affiliations:** 1 Jiangsu Key Laboratory of Crop Genetics and Physiology/Jiangsu Key Laboratory of Crop Cultivation and Physiology/Jiangsu Co-Innovation Center for Modern Production Technology of Grain Crops/Research Institute of Rice Industrial Engineering Technology/Key Laboratory of Saline-alkali Soil Improvement and Utilization (Coastal Saline-Alkali Lands)/Ministry of Agriculture and Rural Affairs, Yangzhou University, Yangzhou 225009, China; 2 Yibang Agriculture Technology Development Co., Ltd., Dongying 257000, China

**Keywords:** *HKT*, salinity, seawater, hormones, rice

## Abstract

The high-affinity potassium transporter (*HKT*) genes are key ions transporters, regulating the plant response to salt stress via sodium (Na^+^) and potassium (K^+^) homeostasis. The main goal of this research was to find and understand the *HKT* genes in rice and their potential biological activities in response to brassinosteroids (BRs), jasmonic acid (JA), seawater, and NaCl stress. The in silico analyses of seven *OsHKT* genes involved their evolutionary tree, gene structures, conserved motifs, and chemical properties, highlighting the key aspects of *OsHKT* genes. The Gene Ontology (GO) analysis of *HKT* genes revealed their roles in growth and stress responses. Promoter analysis showed that the majority of the *HKT* genes participate in abiotic stress responses. Tissue-specific expression analysis showed higher transcriptional activity of *OsHKT* genes in roots and leaves. Under NaCl, BR, and JA application, *OsHKT1* was expressed differentially in roots and shoots. Similarly, the induced expression pattern of *OsHKT1* was recorded in the seawater resistant (SWR) cultivar. Additionally, the Na^+^ to K^+^ ratio under different concentrations of NaCl stress has been evaluated. Our data highlighted the important role of the *OsHKT* gene family in regulating the JA and BR mediated rice salinity tolerance and could be useful for rice future breeding programs.

## 1. Introduction

Rice is a major agronomic crop, providing energy to nearly 2.7 billion people [1]. China is the leading rice producer, followed by India, Indonesia, Bangladesh, and Vietnam. Since it is grown in different agro-zones, variations in size, color, and gene pool are common [2]. However, the looming threat of climate change has hampered rice productivity greatly in the last decade. Among the various environmental stresses, salinity is the most important and concerning abiotic stress impacting the livelihoods of rice growers around the globe [3,4]. The International *Oryza* Map Alignment Project, a wild rice genomic resource, provided a cultivated rice (*O. sativa)* genomes (3K-RG) dataset aiming to catalog natural variations that exist in cultivated and wild rice. The information can be utilized to identify genes and genomic regions that can be used to drive the next generation of crops in the era of climate change [4,5].

About 45 million hectares of the total arable land have been affected by salinity stress (http://www.fao.org/soils-portal/en/) (accessed on 5 April 2022). Salinity damages the rice plant at the seedling and vegetative stages; and salt’s occurrence during the reproductive phase can significantly limit the rice yield [6]. Rice plants respond to salinity stress by adjusting the balance of Na^+^ ions in roots and shoots [7,8]. These sodium ions are regulated by an ion transporter family called the high-affinity *HKT* gene family [9]. Based on gene organization, protein structure, and ion specificity, *HKT* transporters are divided into two sub-types: sodium uniporters (type I) and sodium/potassium symporters (type II). The selectivity filter of type I and II *HKTs* differs significantly [10], providing ion specificity. The key differentiating feature of the *HKT* protein’s first pore domain (PD) is an amino acid [11]. The SF of type I members has a serine residue, whereas the remaining three amino acid residues are glycine (S–G–G–G motif). At all points in the SF, type II *HKTs* exhibit the G–G–G–G motif. However, this rule has exceptions, and extra residues are implicated in providing cation selectivity [12]. In Arabidopsis, there is just one gene, *AtHKT1* [13]. Both dicot and monocot species have large numbers of *HKT* genes [14]. In both *A. thaliana* and cereal crops (particularly type I *HKT1; 5 s*), allelic variants related to amino acid changes and/or expression differences have been linked to conferring salt tolerance [15]. *HKT1; 5* transports Na^+^ across the plasma membranes, decreasing xylem sap (loaded/transferred to neighboring parenchyma cells) and Na loading, and transporting Na^+^ to the shoot. Introduction of *Triticum monococcum* Nax2 (*TmHKT1; 5-A*) into salt-sensitive durum wheat (lacking *HKT1; 5-A*) reduces leaf Na salty soils and increases the grain yield [16]. In bread wheat, the *KNA1* locus (which contains *TaHKT1; 5-D*) is linked to salinity tolerance, and *RNAi* silencing of *TaHKT1; 5-D* in transgenic bread wheat causes leaf Na^+^ accumulation [17]. Salinity tolerance is linked to allelic variation in *OsHKT1; 5* at the Saltol locus in rice, and four amino acid variations in the *OsHKT1; 5* sequences impart differential Na+ transport characteristics [18]. Variations in *OsHKT1; 5* expression levels in rice landrace roots are also linked to salinity tolerance [19]. Two SNPs in the coding region *ZmHKT1; 5* in maize are strongly linked to salt tolerance variants [9]. Natural variation in *HvHKT1; 5* expression levels has recently been linked to salt exclusion from barley shoots. They researchers found that functionally compromised alleles of *HvHKT1; 5* (owing to miss localization and/or altered Na^+^ transport activity) increase shoot and grain Na^+^ content. As a result, allelic variation in *HKT1; 5* may influence shoot Na^+^ content, and as a result, plant fitness in salinity [20,21,22]. Rice crops’ evolutionary diversity allows them to adapt to harsh environmental conditions through specialized molecular mechanisms. However, the exact molecular mechanisms of those adaptations are poorly understood.

Herein, we identify the HKT gene family in *Oryza sativa*. Bioinformatic analysis, including of the phylogenetic tree, conserved motifs, gene structures, gene ontologies, *cis*-acting elements, and microarray expression, was performed. Their expression levels in different organs and tissue were also investigated. Expression analysis of the HKT gene family under jasmonic acid, brassinosteroids, seawater, and salinity stress was evaluated. The results might be helpful for understanding the role of HTK in plant development and elucidating its regulatory mechanisms that are involved in abiotic stress responses.

## 2. Materials and Methods

### 2.1. HKT Genes Family Identification

We used the hidden Markov model (HMM) to retrieve the protein sequences of *HKT* genes from *Arabidopsis thaliana* [23] and *Triticum aestivum* [24]. CD-search NCBI (http://www.ncbi.nlm.nih.gov/Structure/cdd/wrpsb.cgi) (accessed on 6 February 2022) was used to analyze the extracted *Oryza sativa* protein sequences.

### 2.2. Phylogenetic Tree and Digital Expression

The Mega (version 7.0) (Bellingham Research Institute, Bellingham, WA, USA) program created the maximum likelihood phylogenetic tree [25]. The gene expression levels were determined at various stages in all available tissues. The RNA-seq data were retrieved in transcripts per million (TPM) from the expVIP rice Expression Browser (http://www.rice-expression.com/) (accessed on 8 February 2022) [26,27]. The abiotic stress comprises salinity stress, whereas hormonal treatment comprised jasmonic acid (JA) and brassinosteroids (BRs) application. The ratios of the expression levels under different treatments compared to the control level were calculated to determine the regulation patterns of a given gene subjected to stress. Ratios greater than or less than 1.0 under a given treatment indicated that the stress treatment had altered gene expression. In contrast, a ratio of 1.0 showed that the treatment did not affect gene expression [27]. The heatmap was created using the Heml 1.0 software tool (http://hemi.biocuckoo.org/faq.php) (accessed on 8 February 2022) [28].

### 2.3. Chromosomal Locations and Protein–Protein Interactions of OsHKT Genes

The chromosomal locations of the *OsHKT* genes were determined using plants from the Ensemble Genomes (http://www.ensemblegenomes.org/pub/plants/release-31/fasta/oryzasatviam/) (accessed on 10 February 2022) [27]. The MAPD raw was also used to map the physical locations of *OsHKT* genes, and nomenclature was assigned according to the order in which they appeared on the chromosomes. Investigations into rice protein–protein interactions were conducted using the STRING online server (http://string.embl.de) (accessed on 10 February 2022) (version. 10) [28].

### 2.4. Gene Structure and Conserved Motif Analysis

For the Gene structure analysis, Gene Display (http://gsds.cbi.pku.edu.cn/) was utilized by using the genomic and CDS sequences of *OsHKT* genes [29]. The conserved motifs in the *OsHKT* proteins were discovered using the online server MEME 4.11.3 (http://meme-suite.org/tools/meme) (accessed on 10 February 2022) [30].

### 2.5. Gene Ontology and Cis-Elements Analysis of HKT Family Genes

A 1.5 Kb genomic DNA sequence upstream of each identified *OsHKT* gene’s start codon was obtained from the Ensemble Plants database (http://plants.ensemble.org/Oryzasativa) (accessed on 11 March 2022) [31]. The online Plant CARE (http://bioinformatics.psb.ugent.be/webtools/plantcare/html/) (accessed on 11 March 2022) database was used to identify *cis*-regulatory elements for all the *OsHKT* genes. Ontology analysis of the *OsHKT* protein sequences was performed using Blast2GO version 2.7.2 (http://www.blast2go.com) (accessed on 11 March 2022), and the groups of GO classification (molecular functions, biological process, and cellular components) were documented [28].

### 2.6. RNA Isolation and cDNA Synthesis

From the stress-exposed seedlings at selected time points, including 0 (control), the roots, leaves, and stems had their total RNA extracted using Trizol reagent (Invitrogen, Carlsbad, CA, USA). First, the DNA was removed using DNase I. The concentration and purity were measured with a NanoDrop 1000 spectrophotometer (Thermo Fisher Scientific, Rockford, IL, USA), and the integrity was checked using 1.5% agarose gel electrophoresis. Following the manufacturer’s instructions, 1 μg of total RNA was reverse-transcribed on a thermocycler programmed at 37 °C for 15 min using the PrimeScriptTM RT Master Mix (TaKaRa, Dalian, China) containing oligo dT primer [28].

### 2.7. Expression Profiling of HKT Genes in Oryza sativa

Two μL aliquots of cDNA were amplified by qPCR in 20 μL reaction volumes using the SYBR Premix Ex TaqTM II (TaKaRa, Dalian, China). The cDNAs were amplified at 95 °C for 2 min, followed by 35 cycles of 10 s at 95 °C, 30 s, and 72 °C for 30 s; and a final extension step of 72 °C for 10 min in a CFX96 real-time PCR system (Bio-Rad Co., Ltd., Hercules, CA, USA). mRNA amounts of all genes were separately quantified with the stable expression of the constitutive reference gene, actin. The specific primers are detailed in (Supplementary Materials, Table S1). After amplification, target gene cycle threshold (Ct) values were normalized to the reference gene by the 2_−ΔΔCT_ method [32]. The data mean values of three biologically independent replicates were used for the final graphs, following the protocol of Ahmad et al. [28].

### 2.8. Na^+^, K^+^, and Their Ratio in Tolerant (3Y9H) and Sensitive (JLY252) Cultivars

#### 2.8.1. Experimental Method

The experiment was conducted in 2021 at the hydroponic test base in the College of Agriculture, Yangzhou University. The hydroponic pond is 580 cm long, 142 cm wide, and 45 cm deep. Each board has 14 planting holes, each with an aperture of 4 cm and a hole spacing of 10 cm. The hole spacing between the two boards is 16 cm. Field seedlings were sown on May 16 and transplanted on June 16. When transplanting, select seedlings of the same size were transplanted: 2 plants per hole, and 5 holes for each variety. A total of 5 salt concentration treatments were set up for this test (0, 25, 50, 75, and 100 mM); this ratio is the quality ratio), and the salt used for testing was industrial salt. The whole nutrient solution of the hydroponic pool consisted of Epsino nutrient mixed with an Arnon traces nutrient solution. The whole nutrient solution was used during transplanting, 1/2 of the whole nutrient solution was used 20 d after transplanting, and 1/4 of the whole nutrient solution was used after ear extraction. A canopy at the top of the pool was fully enclosed to avoid the rain. We adjusted the pH value with dilute sulfuric acid daily to keep it at about 5.5. Pumps were used to oxygenate, to maintain the uninterrupted flow of the nutrient solution. Each hydroponic pool site’s nutrients, salt, and pH were maintained simultaneously, and disease and pest control were carried out at the appropriate times [33].

#### 2.8.2. Measurements of Na^+^, K^+^ Ratio

Plant roots, leaves, and stems were selected for the experiment, and three replicates were picked of each treatment. The samples were dried at 85 °C in the oven for 48 h. Then, the dried samples were ground into a fine powder, and three replicates of 0.445 to 0.460 g were weighted from each sample. Then, the weighted dry powder was digested using the MARS-6 microwave digestion system (applied to each sample 5 mL HNO_3_, 3 mL double-distilled water, and two drops of H_2_O_2_) and prepared for the micro and macronutrient analysis following the methods described by [34,35]. Each sample was filtered with Whatman filter paper (0.45 µm) and then stored in 10 mL plastic tubes before we used it for further analysis. The digested filtrate was used to measure the total Na^+^, K^+^, and their ratio through inductively coupled plasma atomic emission-spectrometry (Model iCAP 6300, Thermo fisher scientific, ICP Spectrometer, Waltham, MA, USA).

### 2.9. Statistical Analysis

The data presented in this paper were analyzed using SPSS software (version 25.0, SPSS Inc., Chicago, IL, USA) for statistical analysis (ANOVA) and statistical significance, and we used the 95% confidence interval (*p* ≤ 0.05). The data are expressed in the form of mean ± standard deviation (SD) of three biologically independent replicates for all measured parameters; and finally, GraphPad Prism (version 8.0.2) (GraphPad Software, Inc., LA Jolla, CA, USA) was used for graphical representations.

## 3. Results

This in the current study, seven *OsHKT* genes from rice were retrieved using the Ensemble Plants database (http://plants.ensemble.org/Oryzasativa) (accessed on 20 April 2022) and were named according to their chromosomal positions, namely, *OsHKT*1, *OsHKT*3, *OsHKT*4, *OsHKT*6, *OsHKT*7, *OsHKT*8, and *OsHKT*9 (Table 1). Additionally, various other features of Oryza sativa *OsHKT* proteins were identified, such as chromosomal coordinates, molecular weights, chemical properties, and isoelectric points (PI), which are listed in Table 1.

### 3.1. Phylogenetic Analysis of OsHKT

**Figure 1:**
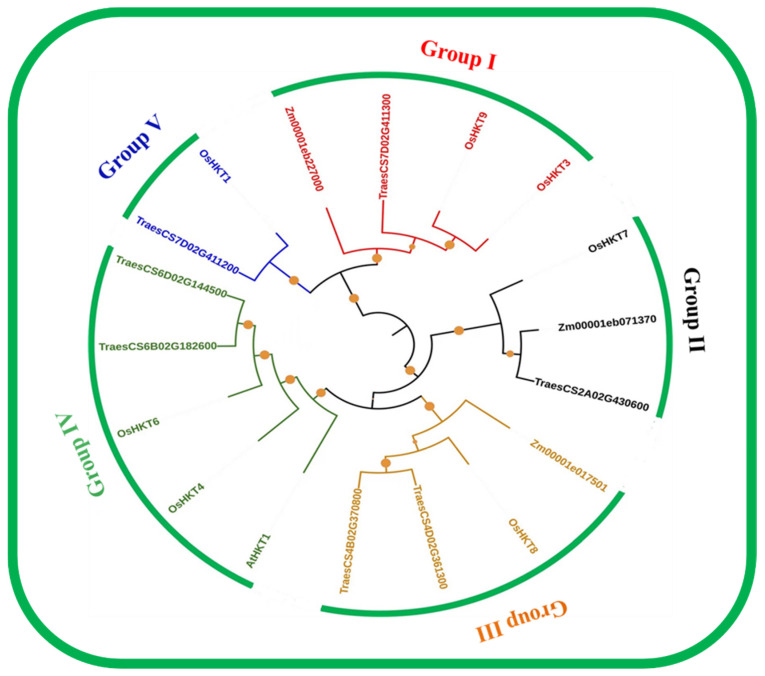
*HKT* protein phylogeny in three plants: *T. aestivum, Zea maize, and O. sativa*. MEGA 7 was used for the phylogenetic tree using the following parameters: Bootstrap D 1000 replicates, maximum likelihood method, and Poisson correction. All group members were divided into five groups, each represented by an assorted color. Different labels were used to identify members of various species.

We used the maximum likelihood method to construct a phylogenetic tree that included *Oryza Sativa OsHKT*, *Triticum aestivum TaHKT*, and *Zea maize ZmHKT* to investigate their phylogenetic relationship, and the final tree was programmed using Interactive Tree Of Life (iTOL) (version 5) (accessed on 25 April 2022) [36] (Figure 1). The results showed that 7 *Oryza sativa OsHKT*, 3 *Zea maize ZmHKT,* and *7 Triticum aestivum TraesHKT* are clustered; and they were further divided into five groups, namely, group I, group II, group III, group IV, and group V. Furthermore, group IV is the largest group containing 2 *OsHKT*, (*OsHKT4* and *OsHKT6*), 2 *TraesHKT*, and 1 *AtHKT.* Group V is the smallest group, containing 1 *OsHKT (OsHKT1)* and 1 *TraesHKT*. Group I contains 2 *OsHKT*, (*OsHKT3* and *OsHKT9*), 1 *ZmHKT*, and 1 *TraesHKT.* Similarly, group III contains 1 *OsHKT* (*OsHKT8*), 1 *ZmHKT*, and 2 *TraesHKT.* Group II contains 1 *OsHKT*, (*OsHKT7*), 1 *ZmHKT*, and 1 *TraesHKT*. These results confirm that evolutionarily, *Zea maize*, *Triticum aestivum*, and *O. sativa* are closest.

### 3.2. Gene Structure and Conserved Motif Analysis of OsHKT Genes

**Figure 2:**
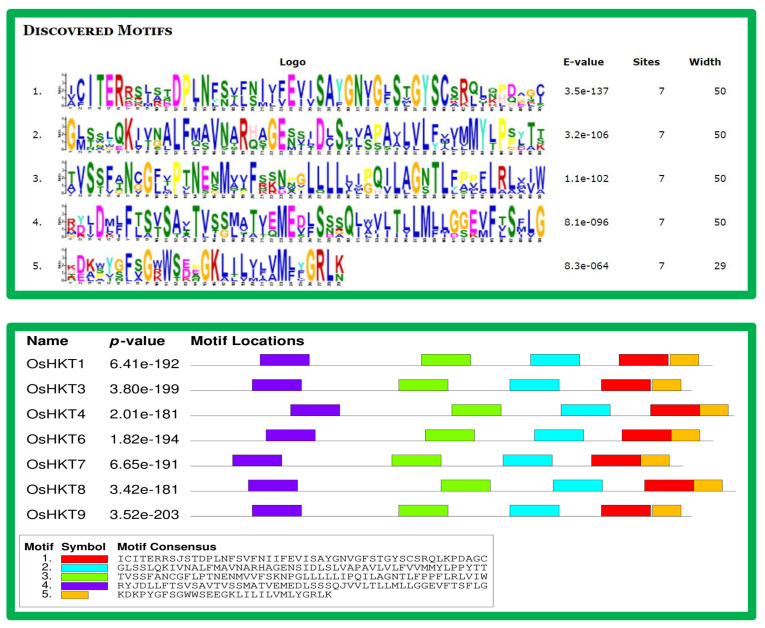
Conserved motifs analysis of *OsHKT* genes in rice. The motif locations and logo are presented separately. The sequences of 5 conserved motifs are presented in the box called Motif Consensus.

A total of five conserved motifs were discovered using the MEME online server (accessed on 5 May 2022), and they were found to be appropriate for explaining the *HKTs* structure (Figure 2). All seven *OsHKT* genes contained all six conserved motifs, motif 1 to motif 6. This result indicates that all motifs are conserved and might play important roles in regulating the expression of the *HKT* gene family. All *HKTs* contained five motifs, and the E-value was <b12 for all identified *HKTs*.

**Figure 3:**
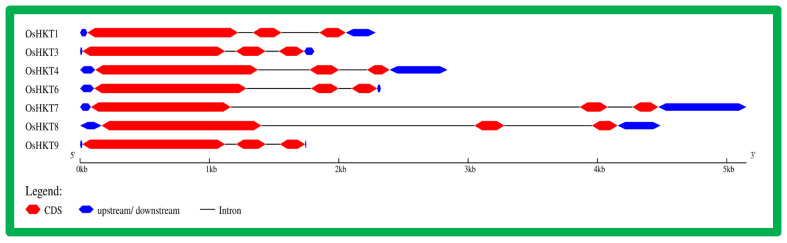
Gene structure analysis of *OsHKT* genes in rice. The red bars show CDS regions, whereas the grey lines represent introns. The blue bars at both ends display upstream/downstream regions.

The genes *OsHKT7* and *OsHKT8* contain more introns as compared to other *HKTs*. In molecular biology, the intron distribution plays a significant role in the phylogenetic relationship among members of the gene family. Our results indicated that *Oryza sativa* is relatively complex, and each *HKTs* gene contains an intron. From the perspective of gene length, *OsHKT 7* and *OsHKT8* are longer than other genes (Figure 3).

### 3.3. Gene Ontology of OsHKT Genes in Rice

**Figure 4:**
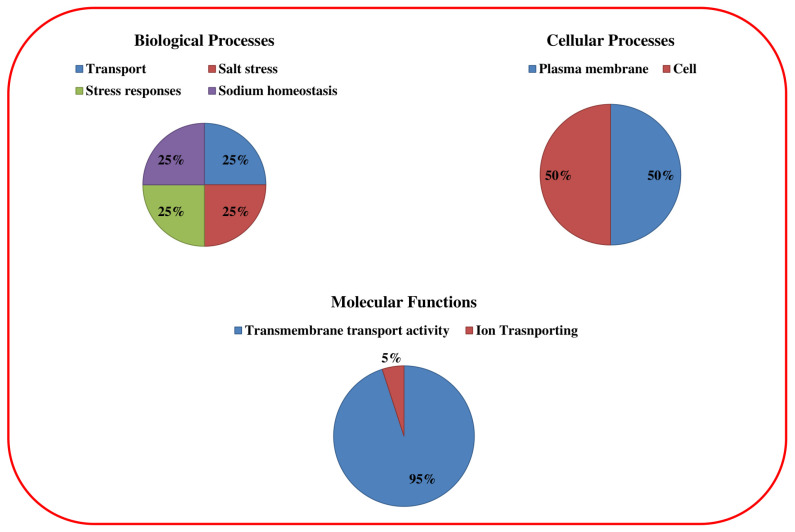
Gene ontology of the *OsHKT* gene family in rice. The figure displays the predicted biological, molecular, and cellular functions of *OsHKT* genes.

Gene Ontology (GO) analysis was performed to explore the putative (biological, molecular, and cellular) functions of the *OsHKT* gene family (Figure 4). According to their molecular functions, *HKT* genes modulate the transmembrane and ion transport activity. Responses to salt stress and sodium homeostasis were revealed by the biological function category. Most of the *OsHKT* genes reside in the nucleus and plasma membrane, revealing their central roles in functional cellular activities.

### 3.4. Cis-Elements of OsHKT Genes in Rice

**Figure 5:**
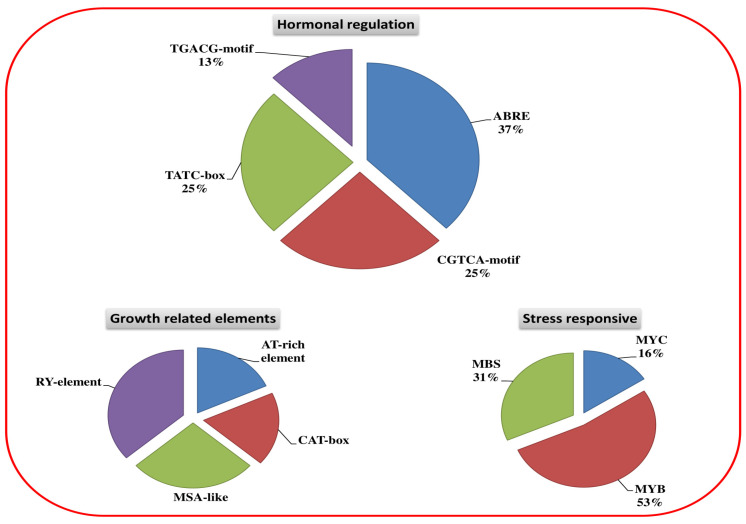
*Cis-*acting elements presented in the 1.5 kb upstream regions of *OsHKT* genes. The figure shows hormonal, stress, and growth responsive *cis*-regulatory elements of the *OsHKT* gene family in rice.

The 1.5 kb upstream regions of the *OHKT* genes were Blasted in the Plant CARE database to identify the *cis-*acting elements. Stress-responsive elements (MYC, MYB, and MBS) were recorded in abundance in *OsHKT* genes (Figure 5). The abscisic acid responsiveness elements (ABRE), gibberellin-responsive elements (TATC box), and MeJA responsive elements (CGTCA and TGACG) were richly represented. Additionally, the CAT box, MSA-like, Ry, and AT rich elements, which are mainly involved in growth-related activities, were identified. Based on these results, it can be assumed that *OsHKT* genes are not only involved in stress biology but also regulate plant growth by fine-tuning the hormonal accumulation.

### 3.5. Protein–Protein Interactions of OsHKT

**Figure 6:**
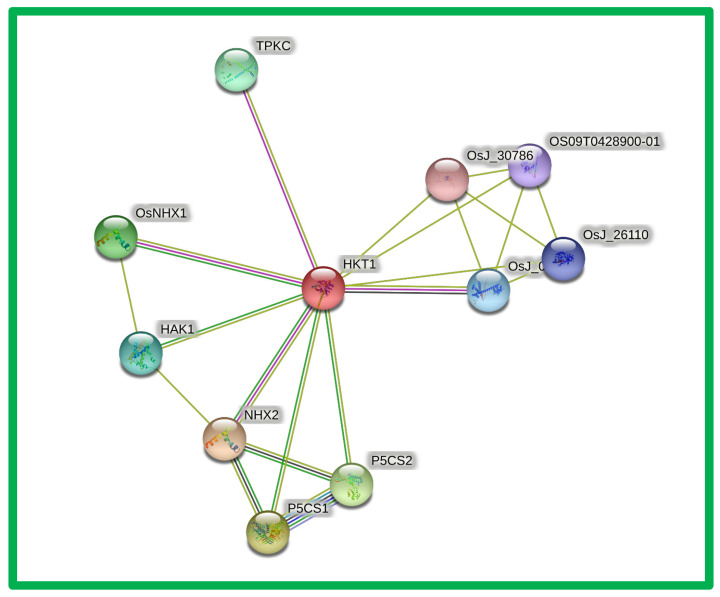
Predictive protein interaction analysis of *OsHKT1* genes in rice. Our reference protein is in the middle in red.

The *OsHKT* protein prediction analysis showed an array of other proteins that have interactions with *OsHKT1* (Figure 6 and Table 2). *NHX2,* which plays a vital role in the putative Na^+^/H^+^ antiporter, a sodium/hydrogen exchanger, showed interaction with our reference gene. Similarly, our gene interacted with *NHX1*, which plays a vital role in sodium/hydrogen exchange; it belongs to the monovalent cation: proton antiporter 1 (CPA1) transporter (TC 2.A.36) family. It also interacted with: *P5CS1,* which plays a vital role in delta-1-pyrroline-5-carboxylate synthase glutamate 5-kinase gamma-glutamyl phosphate reductase; *P5CS* which plays a key role in proline biosynthesis, leading to osmoregulation in plants; *HAK1*, which is potassium transporter 1 and high-affinity potassium transporter, and also transports rubidium with the same affinity. It transports cesium with a lower affinity and belongs to the *HAK/KUP* transporter (TC 2.A.72.3) family. Moreover, our reference gene *OsHKT1* interacts with *OsJ_04382*, which is a putative BTB and TAZ domain protein, and *TPKC*, which is two-pore potassium channel c-containing protein. As an inward-rectifying potassium channel, it belongs to the two-pore domain potassium channel (TC 1.A.1.7) family. The *OsJ_30786* Hsp20/alpha crystallin family protein belongs to the small heat shock protein (*HSP20*) family and also interacts with our reference gene *OsHKT1*.

### 3.6. Tissue-Specific Expression Analysis of OsHKT Genes in Rice

**Figure 7:**
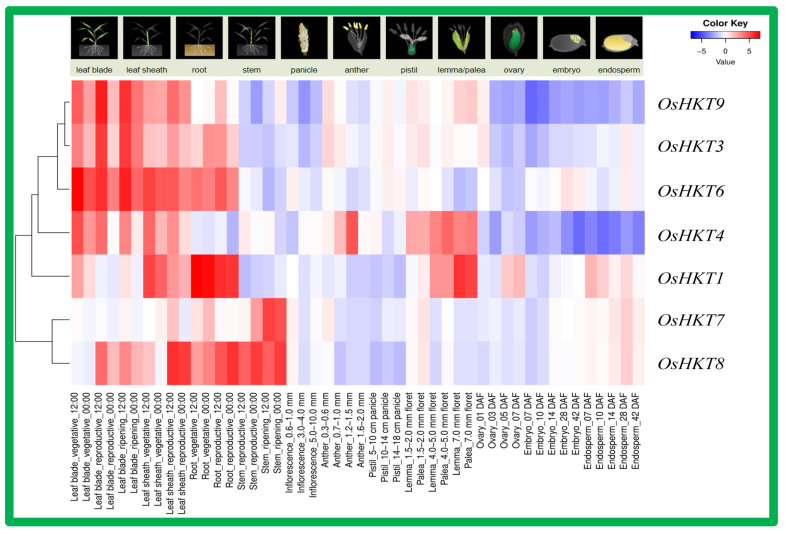
Tissue-specific expression analysis of the *OsHKT* gene family in rice. Data were obtained from a publicly available database. Rows represent *OsHKT* members, and columns show different developmental stages and tissues. The expression level of *OsHKT* [log2 (FPKM  +  1)] is shown by the intensity of color; red represents high expression and blue represents low expression.

Tissue-specific expression is important to understand the functional role of a particular gene family. We analyzed the expression of *OsHKT* genes in different rice tissues by using the RiceXPro expression database (version 3.0, National Institute of Agrobiological Sciences, Tsukuba, Ibaraki, Japan) (accessed on 28 May 2022) [37]. Gene expression patterns in different tissues of rice were drawn on a heatmap, as shown in Figure 7. *OsHKT3*, *OsHKT6*, and *OsHKT9* showed dominant expression in roots, leaf sheaths, and leaf blades. *OsHKT1* displayed higher transcriptional activity mainly in roots. The *OsHKT4* gene was recorded in lamma/palea with higher mRNA accumulation in leaf sheaths, anther, and lamma/palea. The *OsHKT7* exhibited dominant expression in the stem only, whereas *OsHKT8* showed dominant expression in leaf sheaths, roots, and stem tissues.

### 3.7. Expression Analysis of OsHKT Genes in Rice Roots under Brassinosteroids Application

**Figure 8:**
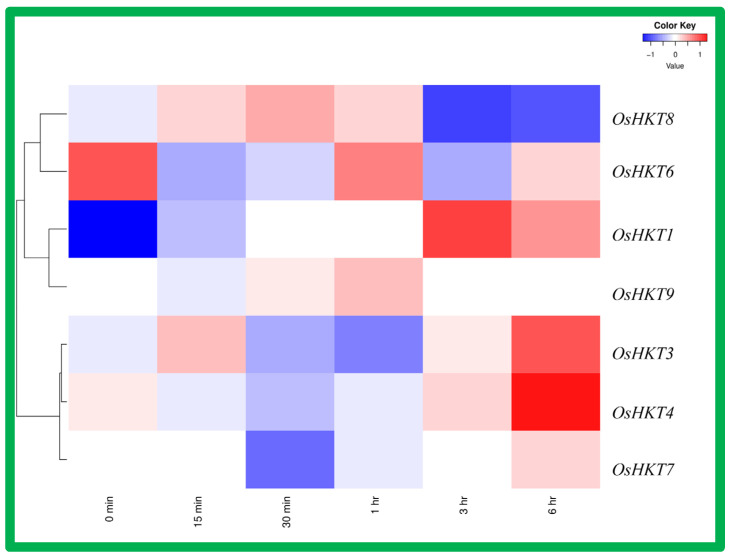
Expression analysis of the *OsHKT* gene family in rice roots under brassinosteroids application. Rows represent *OsHKT* members, and columns show different time points. The expression level of *OsHKT* [log2 (FPKM + 1)] is shown by the intensity of color: red represents high expression, and blue represents low expression.

Brassinosteroids (BRs) are important hormones that regulate plant growth and development. Herein, we show the heatmap of the *OsHKT* gene expression pattern in response to the application of brassinosteroids (Figure 8). Among the *OsHKTs*, *OsHKT3*, *OsHKT4*, and *OsHKT7* showed dominant expression under brassinosteroids application after 6 h of treatment. In comparison, *OsHKT9* expression was lower under various durations brassinosteroid treatment. *OsHKT1* showed lower expression in response to 30 min and 1 h treatments, but higher expression after 3 and 6 h treatments. *OsHKT8* showed high expression initially, (0 min), and as the duration of treatment increased, the expression level also decreased, whereas *OsHKT6* showed dominant expression consistently.

### 3.8. Expression Analysis of OsHKT Genes in Rice Root under Jasmonic Acid Application

**Figure 9:**
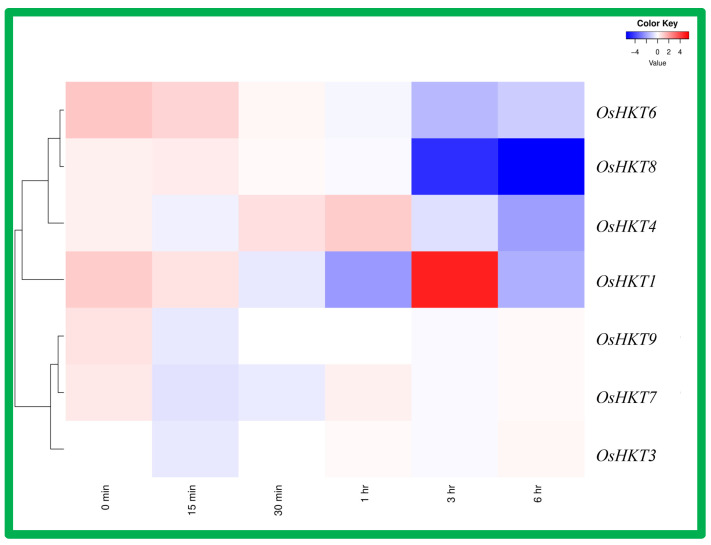
Expression analysis of *OsHKT* genes in rice roots under Jasmonic acid application. Rows represent *OsHKT* members, and columns show different time points. The expression level of *OsHKT* [log2 (FPKM  +  1)] is indicated by the intensity of color: red represents high expression and blue represents low expression.

Gene expression pattern in rice subjected to the jasmonic acid application was drawn on the heatmap shown in Figure 9. The results show that only *OsHKT1* had high expression in response to jasmonic acid treatment after the 3 h duration, and after 6 h duration. *OsHKT8* also displayed relatively high expression as compared to other genes. *OsHKT3*, *OsHKT4*, *OsHKT6*, *OsHKT7*, and *OsHKT9* expression was relatively low as compared to *OsHKT1* and *OsHKT4*.

### 3.9. Expression Analysis of OsHKT Genes in Rice Roots and Stems under Saline Conditions

**Figure 10:**
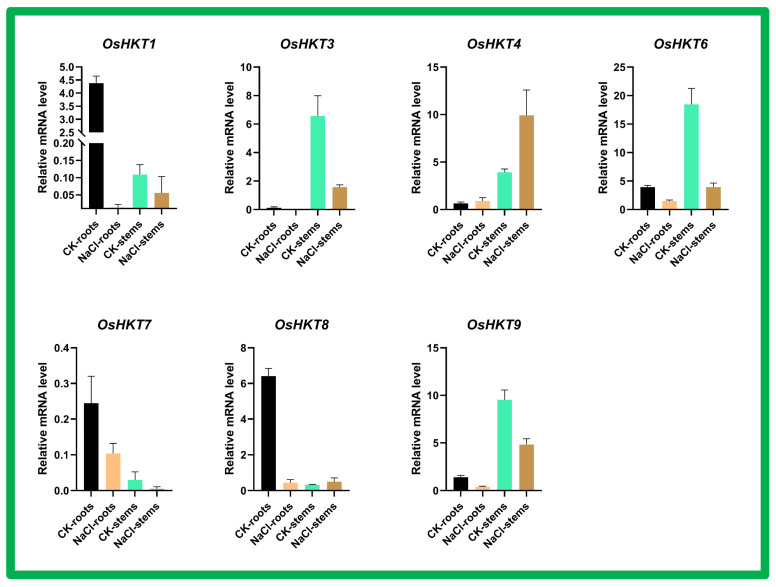
Expression analysis of the *OsHKT* gene family in rice roots and stems under saline conditions. Different treatments were represented with different colors. ANOVA was used to test significance. *p*  <  0.05. Error bars represent standard deviation.

Based on the bioinformatically predicted data, we further studied the response of *OsHKT* genes in rice roots and stems under saline conditions (Figure 10). *OsHKT1* is down regulated, and no expression was recorded in NaCl roots, whereas control CK roots showed high expression. Similarly, in stems, *OsHKT1* transcript level was low under NaCl treatment compared to CK stems. The *OsHKT3* transcript level was lower in roots. High expression was recorded in the stems, and as compared to CK stems, the NaCl treatment showed a lower mRNA level. The *OsHKT4* transcript showed normal expression in roots for both control and treatment, whereas in the stems, the expression level was higher under NaCl treatment as compared to control. The *OsHKT6*, *OsHKT7*, *OsHKT8*, and *OsHKT9* transcript levels were also lower than in the control in both stems and roots.

### 3.10. Expression Analysis of OsHKT Genes in Salinity Resistant Cultivar Subjected to Seawater Stress

**Figure 11:**
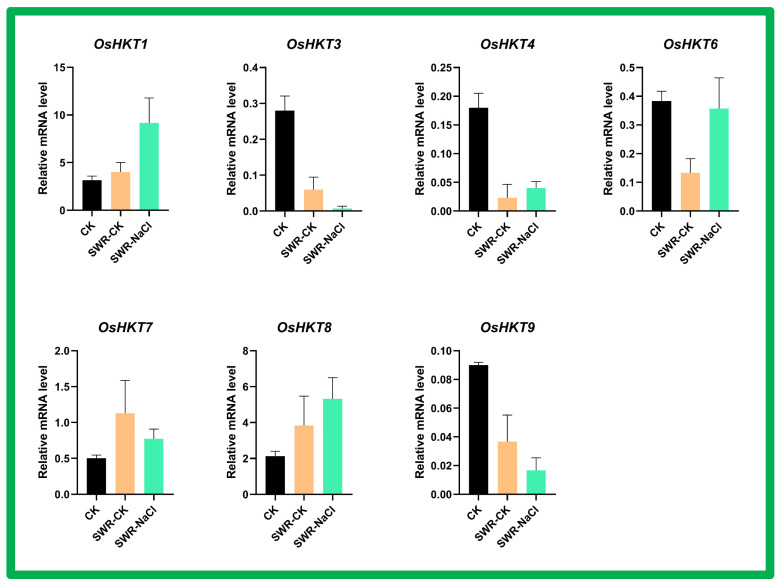
Expression analysis of the *OsHKT* gene family in the salinity resistant cultivar subjected to sea water stress. Control (CK) and seawater resistant (SWR). Different treatments are represented with different colors. ANOVA was used to test significance at *p* < 0.05 probability level. Error bars represent standard deviation.

Based on the bioinformatically predicted data, we further studied the response of the *OsHKT* gene family in the salinity resistant cultivar subjected to seawater stress (Figure 11). The results showed that *OsHKT1* was highly expressed in SWR-NaCl as compared to SWR-CK and CK control. The *OsHKT3* and *OsHKT4* transcript levels were low in SWR-NaCl and SWR-CK and high in the CK control cultivar. The *OsHKT6* transcript level was higher in SWR-NaCl and CK cultivars as compared to SWR-CK cultivars under stress. The *OsHKT7* and *OsHKT8* transcripts showed higher expression levels in SWR-CK and SWR-NaCl cultivars compared to the CK control. The *OsHKT9* transcript level was lower than in the control in both SWR-CK and SWR-NaCl cultivars.

### 3.11. Na^+^, K^+^ Ratio in Tolerant Cultivar 3Y9H

**Figure 12:**
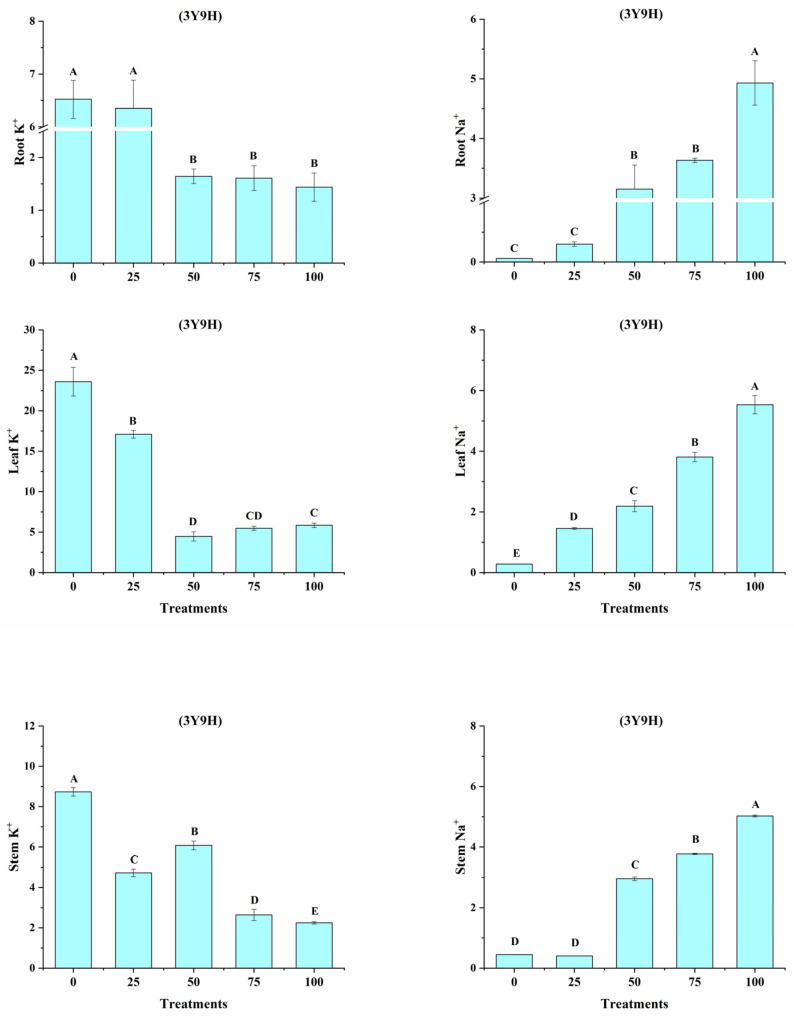
Na^+^, K^+^, and ratio in tolerant cultivar (3Y9H) and five salt concentration treatments (0, 25, 50, 75, and 100 mM). Different letters showed the significant variance at *p* < 0.05.

Based on our results and the functions of *OsKHT* genes, we further tested the 3Y9H cultivar under five salt concentration treatments (25, 50, 75, 100 mM). The tissues tested were the root, leaf, and stem tissues (Figure 12). The results indicate that K^+^ ratio was higher under control and 25 mM conditions, whereas suppression was observed at 50, 75, and 100 mM. In contrast, the ratio of Na^+^ increased under 50, 75, and 100 mM treatments of salt as compared to the control conditions. Similarly, in leaf tissues, the ratio of K^+^ was higher under control conditions as compared to 25, 50, 75, and 100 mM salt treatment. In contrast, the ratio of Na^+^ in leaves increased in 50, 75, and 100 mM treatments of salt as compared to control conditions. In stem tissues, the ratio of K^+^ was higher under control, 25, and 50 mM treatments; however, it declined under 75 and 100 mM treatments. In contrast, the ratio of Na^+^ in leaves increased in 50, 75, and 100 mM treated stem tissues as compared to control tissues.

### 3.12. Na^+^, K^+^ Ratio in the Sensitive Cultivar (JLY252)

**Figure 13:**
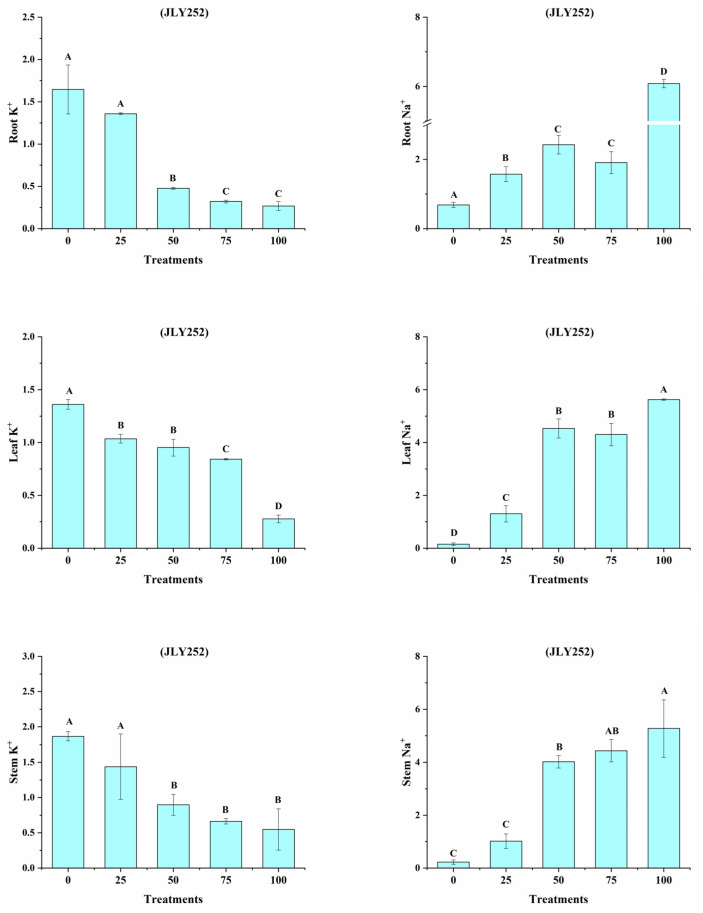
Na^+^, K^+^, and ratio in sensitive cultivar (JLY252) and five salt concentration treatments (0, 25, 50, 75, and 100 mM). Different letters showed the significant variance at *p* < 0.05.

The JLY252 cultivar was subjected to five different salt concentrations (25, 50, 75, 100 mM). The tissues tested were the roots, leaves, and stems (Figure 13). The results indicated that the K^+^ ratio in root tissues was higher under control and 25 mM conditions, and lower under 50, 75, and 100 mM treatments with salt. In contrast, the ratio of Na^+^ in 50 and 100 mM salt treated tissues was higher as compared to 25, 75 mM, and control conditions. Similarly, in leaf tissues, the ratio of K^+^ was higher under control, 25, 50, and 75 mM treatments as compared to 100 mM salt treatment. In contrast, the ratio of Na^+^ in leaves increased under 50, 75, and 100 mM as compared to the control tissues. In stem tissues, the ratio of K^+^ was higher under control, 25 50, and 75 mM treatments, whereas a decreasing trend was recorded for 100 mM. In contrast, the ratio of Na^+^ in leaves increased under 50, 75, and 100 mM conditions as compared to the control.

## 4. Discussion

Most studies on the functional analysis of *HKT* genes have been carried out in model plants, mainly *A. thaliana* [38], and in crops such as wheat [16], barley [39], sorghum [40], and maize [41]. However, a comprehensive study of the rice *HKT* gene family was missing.

### 4.1. OsHKT Genes Are Distributed Widely in the Rice Genome

We identified eight *HKT* genes from the *Oryza sativa* genome database. The *HKT* genes’ classification in different species was confirmed by using the HMM and CD online servers. The absence of a single amino acid residue from the domain indicated the *HKT* genes as main transporters of ions [42]. Based on these results, it can be suggested that these *HKT* genes do not affect the plant’s resistance to salinity in a direct manner. These *HKT* genes could increase plant resistance to salt via balancing the ions’ accumulation in the roots and shoots.

The *HKT* genes in rice are divided in to five subgroups, as showed by the phylogenetic tree analysis (Figure 1). Further, shared homology between the *HKT* genes from each subgroup displays their evolutionarily unchanged cladding. Given the obtained results, the *HKT* genes could be highly conserved and may participate in several other functions (Figure 3). The division of each subfamily also highlighted the diverse functions of these myriad biomolecules in maintaining plant fitness against salinity stress [42,43]. Instead of the series of evolutionary events, the highly conserved grouping of *HKT* genes confirmed that they possess similar functions [44].

We noted that the intron–exon, motif distribution, and physiochemical properties of all HKT proteins were quite similar in each subfamily. For instance, we noted that HKT proteins with more than one intron are classified into subfamily V, and one or intron-less HKT proteins were divided into subfamilies I, II, and IV (Figure 2). Similarly, we also noted that a maximum number of HKT proteins that consisted of five or less motifs were found in subfamily V. The results further noted that the physicochemical properties of all HKT proteins were similar in each subfamily. These results suggest that HKT proteins shared a close evolutionary relationship during biological evolution in plants [45,46]. The intron–exon distribution revealed that most *HKT* genes possessed two exons and one intron (Figure 2), further confirming that the *HKT* genes may have close evolutionary relationships in plants [47]. Furthermore, the motif analysis also noted that all HKT proteins consisted of a total of five to seven motifs (Figure 3), indicating the strong evolutionary relationship of the HKT proteins [48,49]. In addition, the in silico analysis found that the HKT proteins interact with other proteins to regulate the development of different aspects of plant growth (Figure 6).

### 4.2. OsHKT Genes Control Plant Response to Salt Stress, Brassinosteroids, and JA Treatments

Studies have shown that *OsHKT1* is mainly expressed in the shoots, and *OsHKT1;5* is mostly expressed in roots [50]. Our results also confirmed that *OsHKT4* and *OsHKT9* are highly expressed in stem tissues under salt stress (Figure 11). Results have shown that *OsHKT1* is mainly expressed in roots; in contrast, our results showed that *OsHKT1* is mostly expressed in the stem. *OsHKT3*, *OsHKT6*, and *OsHKT9* are mainly expressed in stem cells, and their expression significantly affects ABA induction. This result is in line with previous studies [50,51]. Studies have shown that *HKT1; 5* can take much Na from xylem offloading into surrounding parenchyma cells, reducing xylem SAP. The Na^+^ content prevents transport to the aboveground part of the plant, indirectly raising the content of *K^+^* aboveground [52]. *OsHKT1*, *OsHKT6*, and *OsHKT8* are highly expressed in SWR-NaCl. The results confirmed that these genes play a significant role in salinity stress management.

Previous studies highlighted that *HKT* genes work in concert with other transcription factors. These transcription factors, including MYBc and BHLH, alter the transcriptional activity of *HKT* genes, and thus regulate the plant’s response to salinity stress [53,54,55,56]. For instance, the expression of *OsHKT1* was decreased significantly in the *OsMYBc* antisense lines and in the process compromised the response of rice plants to salinity stress [57]. These multiple lines of evidence suggest the prominent role of *OsHKT* genes in rice tolerance to salinity stress by complex genetic and epigenetic machinery.

Excessive Na^+^ in plants could result in ion toxicity, which causes an imbalance in ion uptake and nutritional disorders and leads to growth inhibition and even plant death [58,59]. Maintaining K^+^ and Na^+^ ion homeostasis is important for a series of physiological and biochemical processes in plants, and for resistance to salinity [60]. In the present study, the concentration of Na^+^ ions was significantly lower in the tolerant line than that in the sensitive line in both leaves and roots. Furthermore, the concentration of K^+^ ions in the tolerant line was significantly higher than in the sensitive line. These results indicate that tolerant lines may have a more selective absorption and transportation capacity for K^+^ and Na^+^ and could maintain a higher concentration of K^+^ under saline conditions.

## 5. Conclusions

We conducted comprehensive bioinformatics and functional analysis of the *OsHKT* gene family in rice. The in silico analysis revealed the potential biological and molecular roles of the *OsHKT* genes in rice growth and stress biology. Expression analysis of *OsHKT* genes revealed their putative role in regulating the rice plant growth under normal and salinity stress conditions. Additionally, *OsHKT* genes showed sensitivity to different hormones, highlighting their importance in hormonal-mediated stress responses and growth regulation. The hydroponic experiment of the two cultivars clearly indicated that the 3Y9H cultivar performed well under the saline condition compared to the sensitive cultivar (JLY252). Higher Na^+^ and low K^+^ content in the sensitive and tolerant cultivars, respectively, further highlighted the potential of HKT transporters in rice’s tolerance against salinity.

## Figures and Tables

**Table 1:** Genomic information of *OsHKT* gene family in rice.

Gene Name	Locus ID	CDS	Chr. Position	AA	MW	PI	SL
*OsHKT*1	LOC_Os06g48,810	1593	Chr6- 29,541,219-29,538,934	531	59,295.0781	9.82610035	PM
*OsHKT*3	LOC_Os01g34,850	1530	Chr1- 19,242,042-19,243,853	510	56,374.3789	9.11009979	PM
*OsHKT*4	LOC_Os04g51,820	1659	Chr4- 30,727,084-30,724,244	553	61,862.4492	8.86629963	PM
*OsHKT*6	LOC_Os02g07,830	1596	Chr2- 4,103,333-4,105,657	532	59,304.5312	9.78820038	PM
*OsHKT*7	LOC_Os04g51,830	1503	Chr4- 30,739,334-30,734,183	501	54,239.3008	8.87370014	PM
*OsHKT*8	LOC_Os01g20,160	1665	Chr1- 11,463,442-11,458,955	555	60,218.2812	8.68159962	PM
*OsHKT*9	LOC_Os06g48,800	1530	Chr6- 29,536,553-29,534,805	510	56,116.7188	8.54440022	PM

AA: amino acid, MW: molecular weight, PI: isoelectric point, SL: subcellular location, PM: plasma membrane.

**Table 2:** Predicted function partners of *OsHKT1* in *Oryza Sativa.*

Reference Protein	Predicted Interactive Partner	Annotation and Putative Function
*OsHKT1*	*NHX2*	Putative Na^+^/H^+^ antiporter; Sodium/hydrogen exchanger.
	*NHX1*	Sodium/hydrogen exchanger; Belongs to the monovalent cation: proton antiporter 1 (CPA1) transporter (TC 2.A.36) family.
	*P5CS1*	Delta-1-pyrroline-5-carboxylate synthase Glutamate 5-kinase Gamma-glutamyl phosphate reductase; P5CS plays a key role in proline biosynthesis, leading to osmoregulation in plants. Involved in abiotic stress tolerance.
	*HAK1*	Potassium transporter 1; High-affinity potassium transporter. Additionally, transport rubidium, with the same affinity and cesium, with a lower affinity; which belongs to the HAK/KUP transporter (TC 2.A.72.3) family.
	*OsJ_04382*	Putative BTB and TAZ domain protein.
	*TPKC*	Two pore potassium channel c; Inward-rectifying potassium channel; Belongs to the two-pore domain potassium channel (TC 1.A.1.7) family.
	*OsJ_30786*	Hsp20/alpha crystalline family protein expressed; Belongs to the small heat shock protein (HSP20) family.

## Data Availability

Data available on the request of the corresponding author.

## Supplementary Materials

The following supporting information can be downloaded at: https://www.mdpi.com/article/10.3390/bioengineering9090410/s1, Table S1: Primers information of *OsHKT* genes for qRT-PCR expression analysis in rice.
