# Supplementary material for: Transcriptome-Wide Analysis Revealed the Potential of the High-Affinity Potassium Transporter (HKT) Gene Family in Rice Salinity Tolerance via Ion Homeostasis
Source: Bioengineering (Basel). 2022 Aug 23;9(9):410. doi: 10.3390/bioengineering9090410 (PMC9495969; doi:10.3390/bioengineering9090410)
Supplement: Supplementary file 1 [file bioengineering-09-00410-s001.zip › bioengineering-1874762-supplementary.pdf]

Article

Transcriptome-Wide Analysis Revealed the Potential of the High-Affinity Potassium Transporter (*HKT)* Gene Family in Rice Salinity Tolerance via Ion Homeostasis

Table S1. Primers information of *OsHKT* genes for qRT-PCR expression analysis in rice.

Genes	Primer sequences
OsHKT1	F: TGAAGCCAAGCAACCCAGAA<br>R: AGGCTGGAAAGTGTCAGAGC
OsHKT3	F: AGAGGCGACTCCCCAAACT<br>R: TGATCATGTATGCCAGCACCA
OsHKT4	F: GGTGCCTCGGATCTATTGGG<br>R: TCAGAGCAAGGAAACCAGCA
*OsHKT*6	F: GTCCTCAAGCCACGAGACAA<br>R: CCAGAGTTGAGCGCTTGAGA
OsHKT7	F: ACCAGGGAATCAGACTGCTC<br>R: CCATATGCACTGACAACTTCGAC
*OsHKT*8	F: CACCCATTCTGGCTCCAACT<br>R: ATCGGCAGAGCTTTCAGCAT
*OsHKT*9	F: AGTGCCTACGGGAACATTGG<br>R: CACCCCGAGAAGCTGTATGG
